# Expression analysis of inflammatory cytokines in patients with rheumatoid arthritis

**DOI:** 10.6026/973206300215020

**Published:** 2025-12-15

**Authors:** Sylvester Noeldoss Lazarus, Harsh V Salankar, Aditya Bhattacharjee, Amrit Podder, Parth Jani, Kanchan Sonone

**Affiliations:** 1 Department of Pathology, American University of Barbados, Wildey, Barbados (Caricom); 2 Department of Pharmacology, N.K.P. Salve Institute of Medical Sciences & Research Centre and Lata Mangeshkar Hospital, Nagpur, Maharashtra, India; 3 Department of Medicine and Surgery, Amrita School of Medicine, Faridabad, India; 4 Department of Physiology, Teerthanker Mahaveer Medical College & Research Centre, Teerthanker Mahaveer University, Moradabad, Uttar Pradesh, India; 5 Department of General Medicine, Government Medical College, Bhavnagar, India; 6 Department of Biochemistry, SMBT IMSRC, Nashik, Maharashtra, India

**Keywords:** Cytokines, disease activity, inflammation, rheumatoid arthritis, synovial fluid

## Abstract

Cytokines, such as TNF-α, IL-1, IL-6, and IL-17, are implicated in disease progression, their exact contribution and potential as biomarkers for diagnosis and treatment monitoring in RA patients require further exploration. Therefore, it is of interest to investigate the expression of inflammatory cytokines (TNF-α, IL-1, IL-6, and IL-17) in patients with rheumatoid arthritis (RA). Cytokine levels were measured in both blood and synovial fluid, revealing significant elevation in RA patients compared to healthy controls. A strong correlation between cytokine levels and disease activity was observed, emphasising their role in disease progression. Data show that cytokines could serve as biomarkers for RA diagnosis and for monitoring treatment. Further research is needed to explore targeted therapies based on cytokine profiles. This research advances knowledge by identifying inflammatory cytokines as key biomarkers for disease activity in rheumatoid arthritis.

## Background:

Rheumatoid arthritis (RA) is a chronic autoimmune disease that primarily affects the joints, leading to inflammation, pain, stiffness, and eventual joint damage. It is a widespread condition, with a significant impact on the quality of life of affected individuals [1]. The disease is characterized by the body's immune system mistakenly attacking the synovial tissue of the joints, leading to chronic inflammation. This condition can affect people of all ages, but it most commonly appears in middle- aged adults, particularly women [2]. The pathogenesis of rheumatoid arthritis involves complex interactions between genetic factors, environmental influences, and the immune system. Inflammation plays a central role in the development and progression of RA, and various inflammatory cytokines have been identified as key players in this process [3]. Cytokines are small signaling molecules that mediate the immune response. They are crucial in regulating the inflammation and tissue damage seen in RA. Among the most significant cytokines involved in RA are tumour necrosis factor-alpha (TNF-α), interleukin-1 (IL-1), interleukin-6 (IL-6), and interleukin-17 (IL-17) [4]. These cytokines promote inflammation in the joints and contribute to the disease's chronic nature [5]. Research has shown that the expression of these inflammatory cytokines is upregulated in the synovial fluid and tissues of patients with rheumatoid arthritis [6]. This heightened expression of pro-inflammatory cytokines leads to an imbalance in the immune response, further intensifying the inflammation [7]. Understanding the role of these cytokines in RA can provide valuable insights into the underlying mechanisms of the disease and can assist in developing targeted therapeutic approaches [8]. Currently, the management of rheumatoid arthritis typically includes the use of nonsteroidal anti-inflammatory drugs (NSAIDs), disease-modifying antirheumatic drugs (DMARDs), and biologic therapies, such as TNF inhibitors. While these treatments have improved the quality of life for many patients, they do not cure the disease. Furthermore, not all patients respond well to these treatments, highlighting the need for a better understanding of the specific cytokine profiles in RA patients [9]. Personalised therapies based on individual patients' cytokine expression patterns could significantly improve treatment outcomes and reduce the overall burden of the disease [10]. Furthermore, cytokine expression can serve as biomarkers to assess disease activity [11]. By monitoring the levels of specific cytokines in the blood or synovial fluid of patients, clinicians can gain valuable information about the severity and progression of the disease [12]. This approach could lead to more precise and timely interventions, reducing joint damage and improving patient outcomes [13]. Therefore, it is of interest to determine the role of inflammatory cytokines in the pathogenesis of rheumatoid arthritis and to explore potential biomarkers for better diagnosis and treatment strategies.

## Methodology:

This study aimed to analyze the expression of inflammatory cytokines in patients diagnosed with rheumatoid arthritis (RA). The methodology was designed to gather both qualitative and quantitative data on cytokine levels and their relationship to disease severity. The following steps outline the process used in this research. The study involved a cohort of adult patients diagnosed with rheumatoid arthritis according to the 2010 American College of Rheumatology (ACR) criteria. The participants were selected from a hospital's rheumatology department, ensuring that a diverse group of individuals were included, based on age, gender, and disease duration. A control group of healthy individuals, matched for age and sex, was also included for comparison. Blood and synovial fluid samples were collected from all participants. Blood samples were drawn from the antecubital vein, while synovial fluid was obtained from patients who had synovial joint inflammation as a result of RA. The samples were collected under aseptic conditions to prevent contamination. All samples were stored at -80°C until further analysis. The primary inflammatory cytokines investigated were tumor necrosis factor-alpha (TNF-α), interleukin-1 (IL-1), interleukin-6 (IL-6), and interleukin-17 (IL-17). These cytokines were chosen based on their established role in the inflammation associated with RA. The levels of these cytokines were measured using enzyme-linked immunosorbent assay (ELISA), a widely accepted technique known for its sensitivity and specificity. The ELISA kits used were commercially available and validated for human serum and synovial fluid samples. The sample size for this study was determined based on the goal of ensuring statistical power to detect significant differences in cytokine levels between patients with rheumatoid arthritis (RA) and healthy controls. In total, 100 participants were included in the study, with 50 RA patients and 50 healthy controls. The RA patient group was selected to reflect a diverse range of disease severities, while the healthy control group was matched for age, sex, and other demographic factors. This sample size was deemed sufficient to achieve meaningful statistical comparisons and to ensure reliable data for cytokine level analysis. Power analysis was performed prior to the study to estimate the minimum number of participants required to detect a significant difference in cytokine levels with a power of 80% and a significance level of 0.05. The data were analyzed to determine the concentration of each cytokine in both blood and synovial fluid samples. Statistical analysis was conducted to compare the levels of cytokines between RA patients and healthy controls. A t-test was used to assess the significance of differences in cytokine levels between the two groups. Additionally, correlations between cytokine levels and clinical disease measures (e.g., Disease Activity Score 28 or DAS28) were examined.

## Results:

In this study, the expression of inflammatory cytokines TNF-α, IL-1, IL-6, and IL-17 was analyzed in blood and synovial fluid samples collected from patients diagnosed with rheumatoid arthritis (RA) and healthy controls. The goal was to assess whether the cytokine levels were elevated in RA patients compared to the control group and to analyze the correlation between cytokine expression and disease activity. The levels of TNF-α, IL-1, IL-6, and IL-17 were measured in blood samples from both RA patients and healthy controls. The cytokine concentrations were significantly higher in the RA group compared to the healthy control group. Table 1 (see PDF) shows significantly higher levels of TNF-α, IL-1, IL-6, and IL-17 in the blood samples of RA patients compared to healthy controls. Cytokine levels in the synovial fluid were also measured. As expected, the concentrations of TNF-α, IL-1, IL-6, and IL-17 were significantly higher in the synovial fluid of RA patients compared to their blood samples and the controls. Table 2 (see PDF) shows significantly higher levels of TNF-α, IL-1, IL-6, and IL-17 in the synovial fluid of RA patients compared to both blood samples and healthy controls. For RA patients, a direct comparison between cytokine levels in blood and synovial fluid was made. The data revealed a notable difference, with cytokine concentrations in the synovial fluid being much higher than in the blood, suggesting that inflammation in the joints contributes significantly to elevated cytokine levels. Table 3 (see PDF) illustrates a significantly higher concentration of cytokines in the synovial fluid compared to the blood in RA patients. The study also examined the correlation between cytokine levels and the Disease Activity Score 28 (DAS28), a common measure of RA disease activity. A strong positive correlation was found between elevated levels of TNF-α, IL-6, and IL-17 and higher DAS28 scores, indicating that these cytokines play an active role in disease progression. Table 4 (see PDF) shows a significant positive correlation between cytokine levels and DAS28 scores, highlighting their role in disease severity. Finally, the potential of cytokines as biomarkers for RA activity was explored. The data showed that elevated cytokine levels, particularly TNF-α and IL-6, were reliable indicators of active disease and could serve as biomarkers for monitoring disease progression and treatment response. Table 5 (see PDF) demonstrates the sensitivity and specificity of each cytokine as a potential biomarker for RA disease activity. The analysis revealed that inflammatory cytokines, especially TNF-α, IL-1, IL-6, and IL-17, were significantly elevated in both the blood and synovial fluid of RA patients when compared to healthy controls. The highest concentrations were observed in synovial fluid, reflecting the local inflammation in the joints. Additionally, cytokine levels correlated strongly with disease activity, particularly TNF-α and IL-6, which showed the highest correlation with DAS28 scores.The ability of these cytokines to serve as reliable biomarkers for disease activity was also supported by their high sensitivity and specificity, especially TNF-α and IL-6. These findings suggest that cytokine measurement could be useful for monitoring RA progression and adjusting treatment strategies accordingly.

## Discussion:

This study's findings align with and expand upon existing research on the role of inflammatory cytokines in rheumatoid arthritis (RA). The elevated levels of TNF-α, IL-1, IL-6, and IL-17 in both blood and synovial fluid of RA patients corroborate earlier studies highlighting the central role of these cytokines in RA pathogenesis. For instance, a study by Tan *et al.* (2025) [14] observed that miR-146a influences the secretion of TNF-α, IL-6, and IL-17 in RA patients, suggesting a regulatory mechanism in cytokine expression. Similarly, research by Chen *et al.* (2011) [15] demonstrated that anti-TNF- α therapy reduces IL-17 levels in RA patients, indicating the interdependence of these cytokines in disease progression. Furthermore, a study by Sakyi *et al.* (2020) [16] identified elevated intracytoplasmic expression of IL-6 and IL-17A in circulating CD4+ T cells from RA patients, supporting the notion that localised cytokine production contributes to systemic inflammation. The correlation between cytokine levels and disease activity, particularly the strong association between IL-6 and DAS28 scores, is consistent with findings from Wei *et al.* (2015) [17], who reported significant correlations between serum IL-6 and TNF-α levels and RA disease activity. In terms of cytokine profiling, a study by Kondo *et al.* (2021) [18] highlighted the involvement of IL-6, IL-17, and TNF-α in RA pathogenesis, reinforcing the multifaceted role of cytokines in disease progression. These studies collectively underscore the pivotal role of inflammatory cytokines in RA and their potential as biomarkers for disease activity. The consistent elevation of TNF-α, IL-1, IL-6, and IL-17 across different studies supports their central role in RA pathogenesis. The observed correlations between cytokine levels and disease activity further validate their utility in monitoring disease progression and treatment efficacy.

## Conclusion:

The critical involvement of inflammatory cytokines in RA is shown in accordance with known data. The insights gained contribute to a deeper understanding of RA pathogenesis and may inform future therapeutic strategies targeting these cytokines.
